# The structure and vibrational spectroscopy of cryolite, Na_3_AlF_6_

**DOI:** 10.1039/d0ra04804f

**Published:** 2020-07-08

**Authors:** Stewart F. Parker, Anibal J. Ramirez-Cuesta, Luke L. Daemen

**Affiliations:** ISIS Facility, STFC Rutherford Appleton Laboratory Chilton, Didcot Oxon OX11 0QX UK stewart.parker@stfc.ac.uk; Spallation Neutron Source, Neutron Spectroscopy Division, Oak Ridge National Laboratory Oak Ridge TN 37831-6475 USA

## Abstract

Cryolite, Na_3_[AlF_6_], is essential to commercial aluminium production because alumina is readily soluble in molten cryolite. While the liquid state has been extensively investigated, the spectroscopy of the solid state has been largely ignored. In this paper, we show that the structure at 5 K is the same as that at room temperature. We use a combination of infrared and Raman spectroscopies together with inelastic neutron scattering (INS) spectroscopy. The use of INS enables access to all of the modes of Na_3_[AlF_6_], including those that are forbidden to the optical spectroscopies. Our spectral assignments are supported by density functional theory calculations of the complete unit cell.

## Abstract 2

We use a combination of infrared, Raman and inelastic neutron scattering spectroscopies to access all of the modes of cryolite, Na_3_[AlF_6_], including those that are forbidden to the optical spectroscopies.

## Graphical abstract

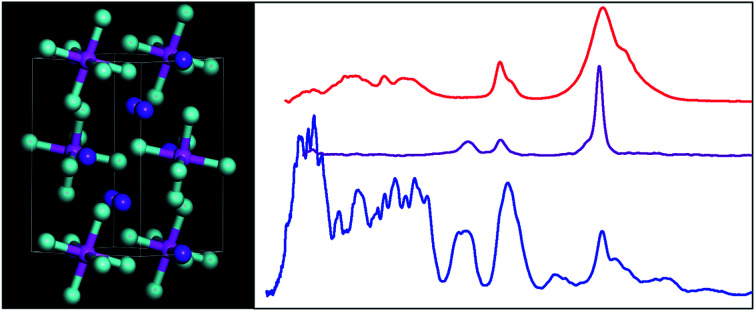

## Introduction

Cryolite, Na_3_[AlF_6_], occurs naturally as a rare mineral.^1^ Historically, it was used as a source of aluminium but this has been superseded by bauxite (a mixture of the Al_2_O_3_ containing minerals boehmite, diaspore and gibbsite), largely because of the higher Al content of bauxite (∼50%) *vs.* cryolite (13%) and the scarcity of the latter. However, cryolite remains essential to aluminium production because alumina is readily soluble in molten cryolite. This is crucial to the economics of aluminium production because cryolite melts at 1012 °C whereas alumina melts at 2072 °C. As the melt is ionic, it also conducts electricity efficiently making the electrolytic reduction of alumina feasible. This is the basis of the Hall–Héroult process, which was invented independently by Hall and Héroult in 1886 and it is still the method of production today.^2^

The liquid phase of cryolite has been extensively investigated by a variety of techniques including multinuclear (^19^F, ^23^Na, ^27^Al) NMR,^3–5^ Raman spectroscopy^6–8^ and quasielastic neutron scattering.^9,10^ There are also a large number of molecular dynamics studies *e.g*.^11–15^, some of which calculate the Raman spectra^13,14^ in the melt. Surprisingly, the solid state has been much less investigated, with only one paper on the infrared spectroscopy of Na_3_[AlF_6_]^16^ and one on that of the isostructural K_3_[AlF_6_].^17^ Cryolite is the end member of the elpasolite family,^18^ the archetype is K_2_Na[AlF_6_], and this is the most abundant prototype in the Inorganic Crystal Structure Database.^19^ The spectroscopy of elpasolite itself has been studied,^20^ as has Cs_2_Na[AlF_6_].^21^ Materials of the type Li_3_[InX_6_] (X = Cl,^22^ Br^23^) are of current interest as lithium ion conductors.^24^

In view of the technological importance of cryolite, we have carried out a comprehensive spectroscopic investigation and report new infrared and Raman spectra over extended temperature and spectral ranges and the inelastic neutron scattering (INS) spectrum. The last of these is observed for the first time and enables access to all of the modes of Na_3_[AlF_6_]. Our spectral assignments are supported by density functional theory calculations of the complete unit cell.

## Results

### Structure

**Fig. 1:**
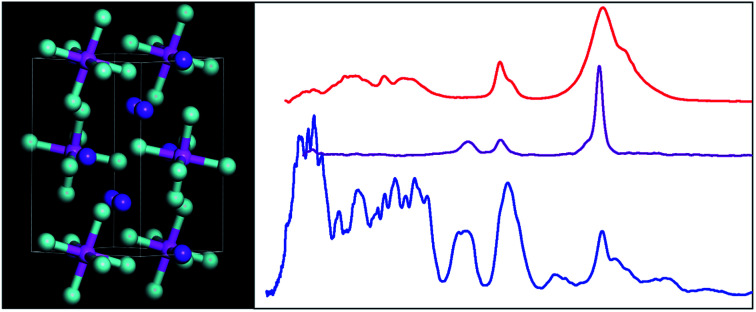
The room temperature structure of cryolite in the monoclinic space group *P*2_1_/*n* (no. 14).^26^ Key: Na1 = orange, Na2 = purple, Al = magenta, F = turquoise.

At room temperature cryolite crystallizes in the monoclinic α-phase, space group *P*2_1_/*n* (no. 14) with two formula units in the primitive cell,^25–27^Fig. 1. The non-standard setting is used because it highlights the relationship to the high temperature (above 823 K) face centered cubic β-phase, *Fm*3̄*m* (no. 225).^27,28^

The structure in the α-phase is shown in Fig. 1 and it can be seen that there are two types of sodium ion: one (Na1, orange) on the Wyckoff site 2c and two (Na2, purple) on the Wyckoff site 4e. These are six- and eightfold coordinated by fluorine atoms, respectively. The [AlF_6_]^3−^ ion is on the Wyckoff site 2d and has *C*_i_ symmetry. Thus the compound is better formulated as: (Na_2_)(Na)[AlF_6_] and is an example of a double perovskite.^27^

**Fig. 2:**
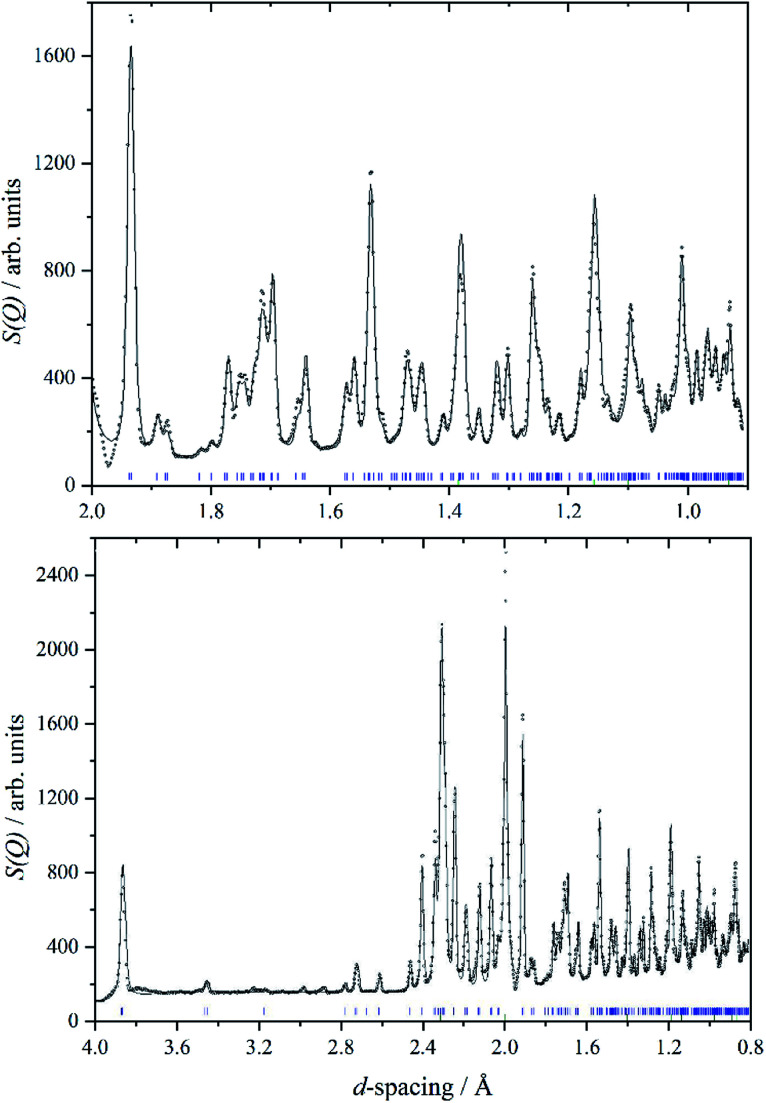
A two-phase Rietveld fit (solid line) to the VISION neutron data (open circles) in the *d*-spacing range 2.0 to 0.8 Å (upper panel) and 4.0 to 0.8 Å (lower panel). Blue tick marks indicate the reflection positions for cryolite, whilst green tick marks indicate the reflections associated with the aluminium sample can. An excellent fit to data is obtained, even though the atomic positions and temperatures factors of the cryolite have not been refined.

We are unaware of any structural studies below room temperature. Heat capacity measurements from 7–350 K,^29^ do not show any evidence of a phase transition in that range, apart from the melting of a liquid inclusion at 268 K in the natural sample of cryolite that was used. However, the INS spectrometer used in this work, VISION,^30^ also has a neutron diffraction capability. Fig. 2 shows a two-phase (cryolite plus the aluminium can) Rietveld fit of the neutron diffractogram measured at 5 K. Scale factors were refined for both phases in obtaining the fit to the data. Lattice parameters have been allowed to refine, to allow for cell contraction, but atomic positions have been held at the room temperature values.^25^ An excellent fit to the data is obtained, even though the atomic positions and temperatures factors of the cryolite have not been refined. Table 1 lists the lattice parameters determined here together with room temperature and estimated values. It is apparent that, apart from the expected lattice contraction on cooling, that the *P*2_1_/*n* structure is retained to at least 5 K.

**Table 1:** Lattice parameters of cryolite

	5^a^ K	0^b^ K	0^c^ K	295^d^ K	295 K^e^
*a*/Å	5.3917(5)	5.381	5.42	5.4139(7)	5.4054(1)
*b*/Å	5.6010(5)	5.581	5.63	5.6012(5)	5.5934(1)
*c*/Å	7.7556(8)	7.693	7.83	7.7769(8)	7.7672(1)
*α*/°	90.000	90.000	90.0	90.000	90.000
*β*/°	90.253(8)	90.285	90.2	90.183(3)	89.81(1)
*γ*/°	90.000	90.000	90.0	90.000	90.000
*V*/Å^3^	234.21(4)	232.6	238.9	235.8	234.84

a This work.

b Extrapolated from ref. 25 by ref. 14.

c Calculated by molecular dynamics.^14^

d From ref. 25.

e From ref. 26.

Table 2 compares selected observed and calculated bond distances (all of the *cis* F–Al–F bond angles are 90 ± 1°, all the *trans* angles are 180° by symmetry). As might be expected from the very small difference between the room temperature and 5 K lattice parameters there is little change in the interatomic distances. The calculation does slightly overestimate the Al–F distances. The Na–F distances are slightly shorter than found in NaF (2.318 Å (ref. 31)).

**Table 2:** Comparison of observed and calculated structure of cryolite

Distance/Å	295^a^ K	5 K initial^b^	5 K opt^c^
Al–F	2 × 1.799	2 × 1.812	2 × 1.825
	2 × 1.820	2 × 1.825	2 × 1.834
	2 × 1.830	2 × 1.836	2 × 1.836
Na1–F	2 × 2.211	2 × 2.205	2 × 2.219
	2 × 2.271	2 × 2.256	2 × 2.257
	2 × 2.272	2 × 2.264	2 × 2.274
Na2–F (min)	2.292	2.287	2.276
(max)	2.816	2.806	2.807
(ave)	2.498	2.494	2.564

a From ref. 26.

b Structure used for the fits in Fig. 2.

c 5 K structure after geometry optimization.

### Vibrational spectroscopy

**Fig. 3:**
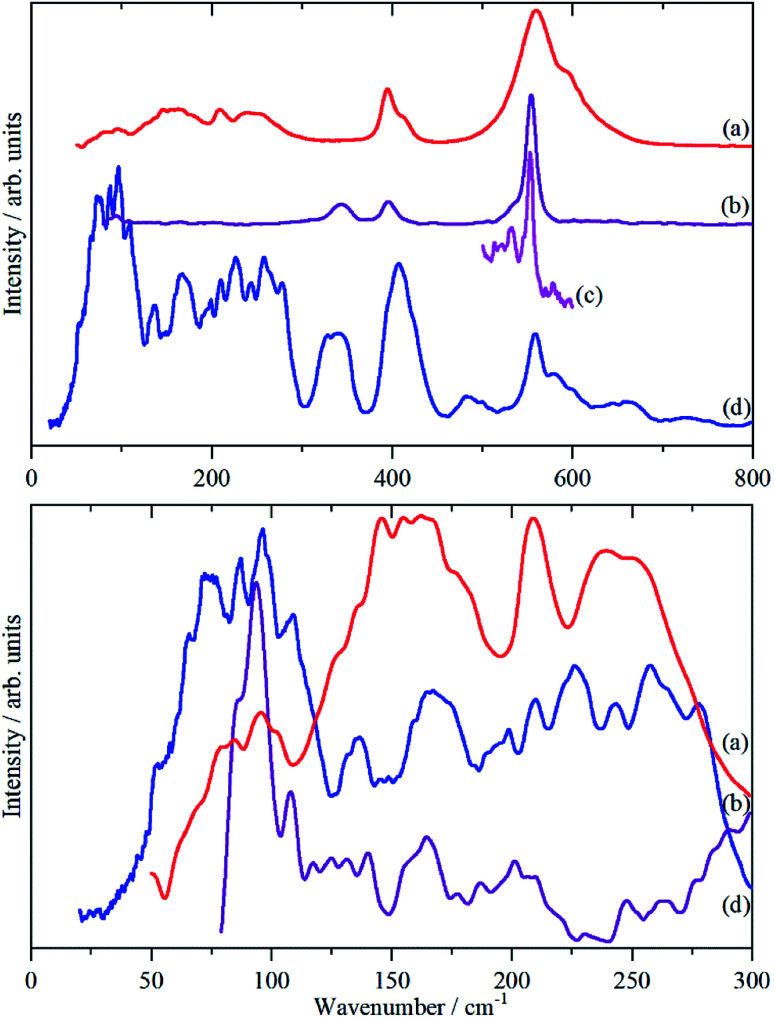
Vibrational spectra of cryolite: (a) infrared at room temperature, (b) Raman at room temperature (1064 nm excitation), (c) Raman at 13 K (785 nm excitation) and (d) INS at 5 K recorded on VISION. The lower panel shows the lattice mode region on expanded scales. Relative to the top panel the spectra are ordinate expanded: (a) ×10, (b) ×40 and (d) ×1.5.

Fig. 3 shows the infrared, Raman and INS spectra of cryolite. The Raman spectrum at 13 K is seen in Fig. 3c, unfortunately, because of sample fluorescence, only the Al–F symmetric stretch mode at 554 cm^−1^ was observable. However, apart from a marked narrowing, there is no shift in transition energy or additional peaks apparent. The lattice mode region, shown in the lower part of Fig. 3, shows coincidences between the INS and the infrared and Raman data. Thus the vibrational spectroscopy suggests (but does not prove) that there is no phase change between room temperature and 5 K, consistent with the diffraction results.

An isolated octahedral, *O*_h_, [AlF_6_]^3−^ ion has six Al–F stretch modes: *ν*_1_ (A_1g_), *ν*_2_ (E_g_), *ν*_3_ (T_1u_) and nine F–Al–F bend modes: *ν*_4_ (T_1u_), *ν*_5_ (T_2g_), *ν*_6_ (T_2u_). *ν*_1_, *ν*_2_ and *ν*_5_ are Raman active, *ν*_3_ and *ν*_4_ are infrared active and *ν*_6_ is forbidden in both forms of spectroscopy.^32^ However, all of the modes are allowed in the INS spectrum. To go beyond this requires more detailed analysis and to this end we use the correlation method.^33^ The results are shown in Table 3.

**Table 3:** Correlation table for cryolite

Ion	*n*	Free ion		Crystal							Factor group	Total^a^
				Site^c^	Translations		Librations		Intramolecular			
		Sym.^b^	Rep.		Rep.	No.	Rep.	No.	Rep.	No.	*C*_2h_	
Na1	2			*C*_i_	A_u_	3					(A_u_ + B_u_)	3(A_u_ + B_u_)
Na2	4			*C*_1_	A	3					(A_g_ + B_g_ + A_u_ + B_u_)	3(A_g_ + B_g_ + A_u_ + B_u_)
AlF_6_	2	*O*_h_	T_1u_	*C*_i_	A_u_	3					(A_u_ + B_u_)	3(A_u_ + B_u_)
	2	*O*_h_	T_1g_	*C*_i_			A_g_	3			(A_g_ + B_g_)	3(A_g_ + B_g_)
	2	*O*_h_	A_1g_ (*ν*_1_)	*C*_i_					A_g_	1	(A_g_ + B_g_)	(A_g_ + B_g_)
	2	*O*_h_	E_g_ (*ν*_2_)	*C*_i_					A_g_	2	(A_g_ + B_g_)	2(A_g_ + B_g_)
	2	*O*_h_	T_1u_ (*ν*_3_)	*C*_i_					A_u_	3	(A_u_ + B_u_)	3(A_u_ + B_u_)
	2	*O*_h_	T_1u_ (*ν*_4_)	*C*_i_					A_u_	3	(A_u_ + B_u_)	3(A_u_ + B_u_)
	2	*O*_h_	T_2g_ (*ν*_5_)	*C*_i_					A_g_	3	(A_g_ + B_g_)	3(A_g_ + B_g_)
	2	*O*_h_	T_2u_ (*ν*_6_)	*C*_i_					A_u_	3	(A_u_ + B_u_)	3(A_u_ + B_u_)

a Total is the product of the column “No.” and the factor group.

b Sym. = symmetry, Rep. = irreducible representation of the point group, No. = number.

c Symmetry of the site occupied by the ion in the crystal.

With two formula units in the primitive cell, there are 20 atoms present hence there are 60 modes, which are given by the sum of the last column in Table 3: 12 A_g_ + 12 B_g_ + 18 A_u_ + 18 B_u_. This includes the three acoustic translational modes, which have A_u_ + 2 B_u_ representations and have zero energy at the Brillouin zone Γ-point, where the infrared and Raman modes are observed. Modes that have A_u_ or B_u_ symmetry are infrared active, those with A_g_ or B_g_ are Raman active. Two deductions are immediately obvious: all of the degeneracies are formally lifted and because the centre of symmetry is preserved, the free ion selection rules will still apply, except that *ν*_6_ is now allowed in the infrared spectrum. As stated earlier, all modes are allowed in the INS spectrum.

The analysis in Table 3 enables some of the modes to be assigned. The intense, broad mode at 559 cm^−1^ with shoulders at 596 and 608 cm^−1^ in the infrared spectrum must be *ν*_3_. Previous work^16^ on the infrared spectrum of cryolite found *ν*_3_ at 599 cm^−1^ with shoulders at 580 and 630 cm^−1^, the lower energy modes were not reported. We can only ascribe the difference in the transition energy of *ν*_3_ to either a calibration error in the older (pre-FTIR) work or that the natural sample of cryolite used there was impure.

The strong mode at 554 cm^−1^ in the Raman spectrum must be *ν*_1_ and the two weaker modes at 396 and 344 cm^−1^ in the Raman spectrum are *ν*_2_ and *ν*_5_ respectively. This is in excellent agreement with the previously reported Raman spectrum of cryolite.^8^ Cryolite melts^6–8^ show a strong band at ∼550 cm^−1^ assigned to *ν*_1_. In the infrared spectrum the mode at 396 cm^−1^ is assigned as *ν*_4_. The coincidence with *ν*_2_ is surprising because one is an Al–F stretch mode and the other an F–Al–F bending mode, but the selection rules are unambiguous. The assignment is supported by the INS spectrum, which shows a mode at 407 cm^−1^, that is much stronger than *ν*_5_, consistent with it being the unresolved sum of the two modes. The optically silent mode *ν*_6_ is not apparent, so it must occur below 300 cm^−1^.

It is only in the region <300 cm^−1^ in the INS spectrum that the low symmetry of the system is readily apparent. In the infrared spectrum, there are three weak features, which Table 3 shows must be *ν*_6_ and the translational modes of the ions, although there is no way to assign which is which. In the Raman spectrum there is a weak mode at 94 cm^−1^, which is presumably the [AlF_6_]^3−^ ion librational mode.

### Computational studies

**Fig. 4:**
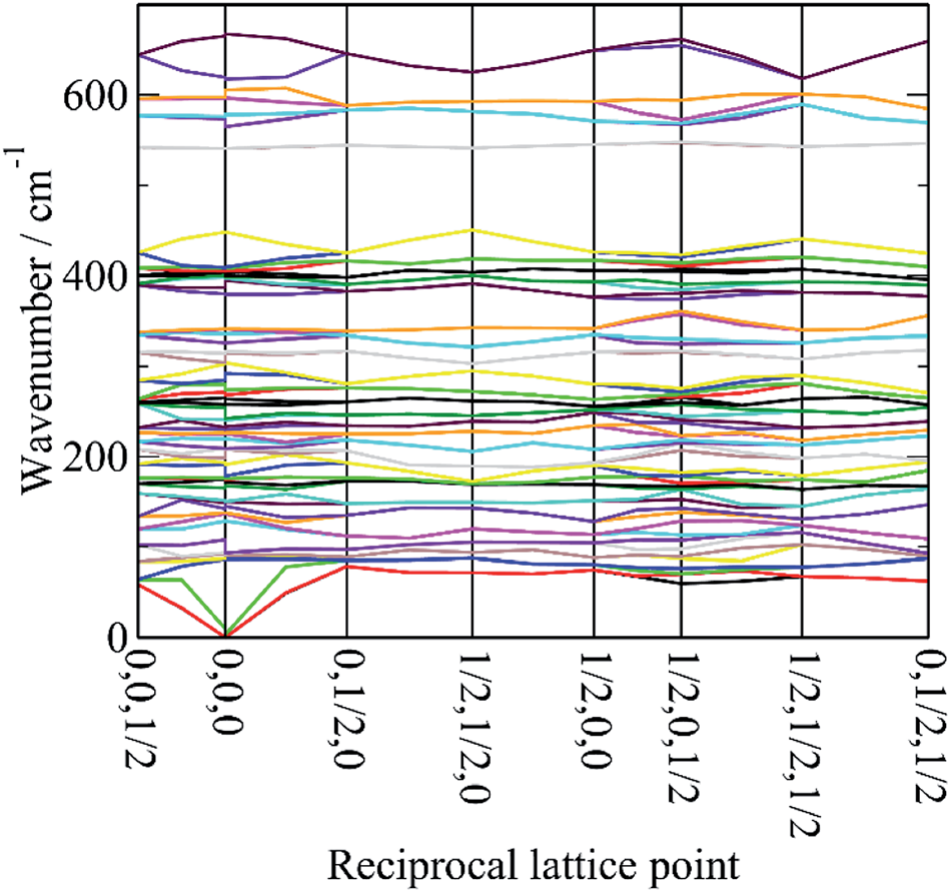
Dispersion curves of cryolite in the monoclinic space group *P*2_1_/*n* (no. 14).

**Fig. 5:**
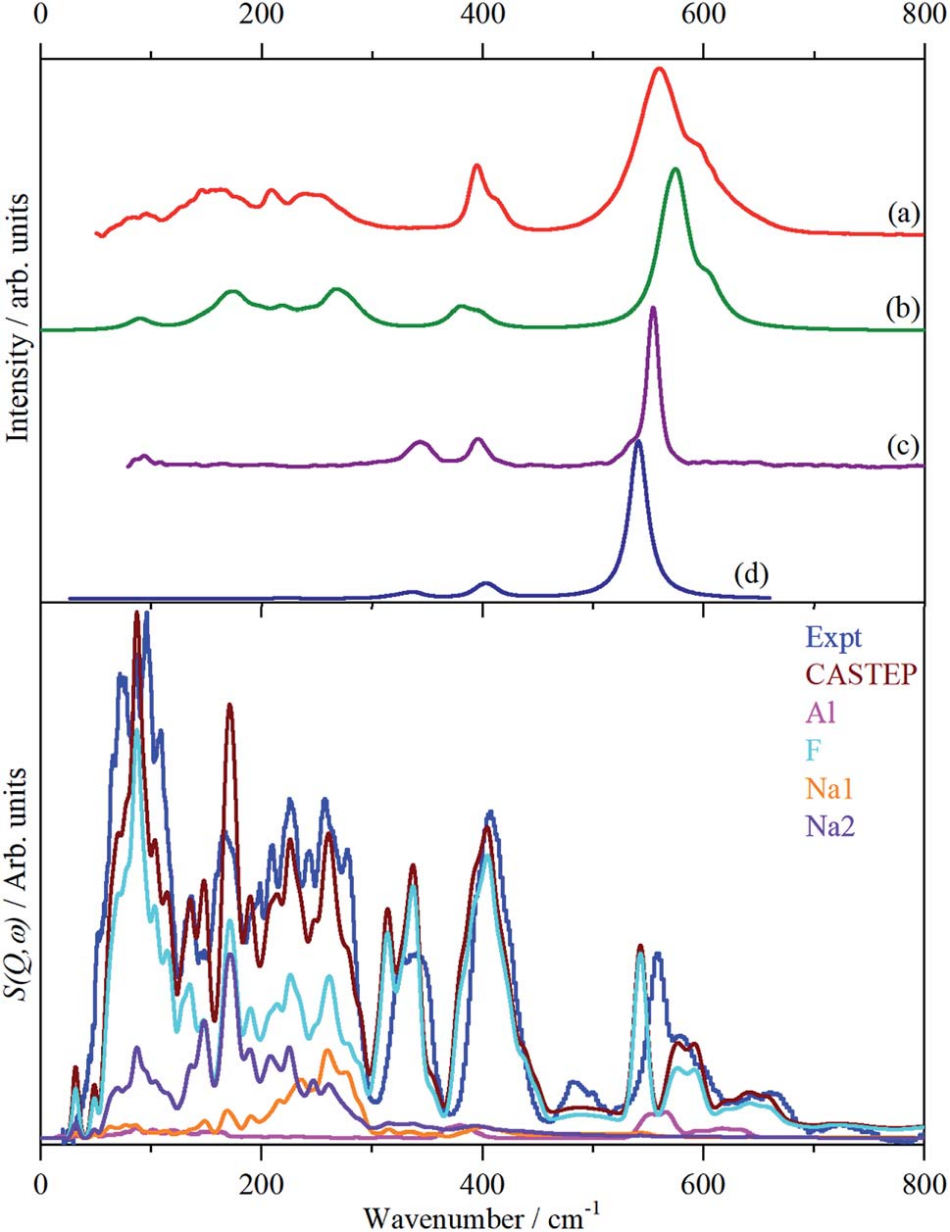
Observed and calculated (CASTEP) spectra of cryolite. Top panel: infrared spectra (a) observed, (b) calculated. Raman spectra: (c) observed, (d) calculated. Lower panel: INS spectra. The individual element contributions to the calculated spectrum are also shown.

In order to assign the low energy region and to confirm the assignments for *ν*_1_ to *ν*_5_ we use periodic density functional theory (DFT) of the primitive cell and calculate the vibrational transition energies across the entire Brillouin zone (see: Materials and methods for details). The resulting dispersion curves are shown in Fig. 4 and the INS spectrum generated from the calculation is shown in Fig. 5.

It can be seen that the calculated INS spectrum is in almost quantitative agreement with the experimental data. The Al–F stretch modes are calculated slightly softer than is observed, this is probably because the bond lengths are calculated slightly too long: observed:^25–27^ 1.799, 1.820, 1.830 Å; calculated: 1.825, 1.834, 1.836 Å. However, the overall pattern of the experimental data is very well reproduced. In particular, the splitting of *ν*_3_ is clearly seen and Fig. 4 shows that this is the result of the combination of the site group and factor group splitting combined with significant vibrational dispersion (variation with wavevector). The dense manifold of modes below 300 cm^−1^ occurs because the low symmetry removes all the degeneracies and results in the complex structure seen experimentally.

The transition energies at the Brillouin zone Γ-point, (0,0,0), are given in Table 4 with the assignments based on visualization of the modes. This confirms the assignments for *ν*_1_ to *ν*_5_ and in particular that *ν*_2_ and *ν*_4_ are coincident. The “missing” mode *ν*_6_ is calculated at 254–288 cm^−1^ and is seen to occur weakly in the infrared spectrum at 239/253 cm^−1^. This is the first time that *ν*_6_ has been observed experimentally.

**Table 4:** Observed and calculated (at the Brillouin zone Γ-point) transition energies with assignments for cryolite

CASTEP/cm^−1^	Sym^b^	IR int/km mol^−1^	Raman int/Å^4^ amu^−1^	Observed^a^			Description^b^
				INS/cm^−1^	Raman/cm^−1^	Infrared/cm^−1^	
0	B_u_	0	0				Acoustic
0	A_u_	0	0				Acoustic
0	B_u_	0	0				Acoustic
86	A_g_	0	0.02	75s			Lib + Na2 trans
87	A_u_	13.62	0				AlF6 trans
90	B_u_	103.00	0				AlF6 trans
92	A_g_	0	0.02	87s	86sh		Lib + Na2 trans
93	B_g_	0	0	96s	94w		Lib + Na2 trans
128	B_g_	0	0	109s	109w		Lib + Na2 trans
137	A_g_	0	0.02	136m			Lib + Na2 trans
138	B_g_	0	0				Lib + Na2 trans
142	A_u_	32.79	0				Na2 trans
149	A_u_	9.73	0				AlF6 trans
150	B_u_	36.73	0			146w	Na2 trans
165	A_u_	55.50	0			154w	Na2 trans
167	B_u_	163.52	0	164s		163w	Na2 trans
175	A_g_	0	0.02				Na2 trans + Lib
175	B_g_	0	0.01				Na2 trans + Lib
178	B_u_	226.57	0			178w	Na2 trans
191	A_u_	5.47	0			183w	Na1 trans
198	A_u_	100.07	0				Na2 trans
209	B_g_	0	0	198w			Na2 trans + Lib
209	A_g_	0	0.02	210m			Na2 trans + Lib
219	A_u_	59.53	0	209m	209w		Na1 trans
219	B_u_	96.45	0				Na1 trans
225	B_g_	0	0.02	226m			Na2 trans + Lib
226	A_g_	0	0.02				Na2 trans + Lib
232	A_u_	1.86	0	243m			Na1 trans
240	B_u_	37.39	0				Na1 trans
242	B_u_	56.98	0			243w	Na1 trans
258	A_u_	1.46	0	257m		252w	*ν*_6_
264	B_u_	199.11	0				*ν*_6_
268	B_u_	73.93	0	265sh			*ν*_6_
271	A_u_	71.91	0				*ν*_6_
280	A_u_	161.49	0	278sh			*ν*_6_
292	B_u_	38.42	0				*ν*_6_
304	A_g_	0	0.01	325s			*ν*_5_
314	B_g_	0	0.07				*ν*_5_
326	A_g_	0	0.18				*ν*_5_
335	A_g_	0	0.24				*ν*_5_
341	B_g_	0	0.16	336s,br	343m		*ν*_5_
341	B_g_	0	0.08				*ν*_5_
379	B_u_	108.53	0				*ν*_4_
380	A_u_	95.40	0				*ν*_4_
397	B_u_	129.31	0	396sh		395m	*ν*_4_
398	A_g_	0	0.62		395m		*ν*_2_
400	B_u_	0.62	0				*ν*_4_
404	A_g_	0	0.36				*ν*_2_
405	A_u_	0.24	0				*ν*_4_
408	B_g_	0	0.50	407s			*ν*_2_
409	A_u_	2.09	0			413sh	*ν*_4_
448	B_g_	0	0.05	425sh			*ν*_2_
541	A_g_	0	13.80				*ν*_1_
541	B_g_	0	0		554s		*ν*_1_
565	B_u_	562.08	0	559s		559s	*ν*_3_
574	A_u_	762.85	0				*ν*_3_
578	B_u_	657.32	0	580m			*ν*_3_
597	A_u_	21.77	0	601m			*ν*_3_
605	B_u_	336.80	0			596sh	*ν*_3_
619	A_u_	21.74	0			608sh	*ν*_3_

a s = strong, m = medium, w = weak, br = broad, sh = shoulder.

b Lib = libration of [AlF_6_]^3−^ ion, AlF6 = translation of [AlF_6_]^3−^ ion, Na1 trans = translational mode of Na1, Na2 trans = translational mode of Na2.

Fig. 5 also includes the individual contributions to the INS spectrum from each element. As expected, the fluorine contribution accounts for most of the intensity and it confirms that the librational modes account for the peaks at ∼85–150 cm^−1^. However, the librations have the same symmetry, A_g_ and B_g_, as some of the translational modes of Na2 which results in extensive mixing of the two vibrations. Thus the lower energy modes around 100 cm^−1^ are more librational in form while the higher energy ones around 210 cm^−1^ are more translational. Na2 has modes of both gerade and ungerade character, Table 3, and this results in the modes occurring in a broad band from 50–300 cm^−1^. In contrast, the Na1 modes are much more localized and largely occur in the narrower range of 200–300 cm^−1^. This is a consequence of the more regular coordination polyhedron of Na1: the Na–F distances vary by less than 0.06 Å, by contrast those around Na2 vary by nearly ten times as much, 0.5 Å (Table 2).

## Discussion

This work provides the first complete assignment of the vibrational spectra of cryolite. In particular, the forbidden (in *O*_h_ symmetry) mode *ν*_6_ has been observed. This is very rare:^32^ (pp. 216–218) lists the spectra of over 30 [MF_6_]^*x*−^ ions; for none of them is *ν*_6_ given, although it is known for a few neutral MF_6_ systems. Assignment of librational modes is equally rare. To our knowledge, the only examples for which this is known are for K_2_[MCl_6_] (M = Pt, Ir,^34^ Re^35^) where the librational transition energies are 55 cm^−1^ (Pt), 48 cm^−1^ (Ir) and 28 and 68 cm^−1^ (Re). Making the naive assumption that it is only the difference in the moment of inertia between the [AlF_6_]^3−^ and [PtCl_6_]^2−^ ions that accounts for the difference in transition energy would predict that [AlF_6_]^3−^ occurs at 87 cm^−1^. This is on the lowest edge of the band of the librational modes (Table 4) and suggests that other factors are also relevant, the most likely being the difference in charge of the ions.

The only unassigned band in the spectra shown in Fig. 3 is the shoulder at 533 cm^−1^ on the low energy side of *ν*_1_ that is clearly resolved at 13 K. We considered the possibility that it may be the B_g_ symmetric Al–F stretch mode that was miscalculated, however, there is no corresponding mode in the INS spectrum, which also argues against it being an overtone or combination. The calculated Raman intensity of this mode is almost zero which also militates against this assignment. Inspection of the Raman spectra of cryolite in the literature^4,6,8^ does not show the band in the solid or liquid phase. This suggests that it is an impurity, despite the stated purity being >99%, and the most likely candidate is chiolite, Na_5_Al_3_F_14_. This mineral occurs naturally with cryolite^36^ and consists of sheets of corner-sharing AlF_6_ octahedra,^37^ it has a very strong totally symmetric Al–F stretch mode at 530 cm^−1^.^38^

## Materials and methods

Cryolite, Na_3_[AlF_6_], (>99%) was purchased from Sigma-Aldrich (Gillingham, Dorset, UK) and used as received.

INS spectra were recorded at 5 K using the VISION^30^ spectrometer at the Spallation Neutron Source (SNS) (Oak Ridge, Tennessee, USA). An empty aluminium sample can has been subtracted from the measured INS data. Infrared spectra (50–4000 cm^−1^, 4 cm^−1^ resolution, 64 scans) were recorded at room temperature with a Bruker Vertex 70 Fourier transform infrared spectrometer using a Bruker Platinum single reflection attenuated total internal reflection accessory. The FT-Raman spectrum was recorded at room temperature a from the sample inside a quartz cuvette with a Bruker MultiRam spectrometer using 1064 nm excitation (500 mW laser power and 1024 scans at 4 cm^−1^ resolution). Variable temperature (13–300 K) Raman spectra were recorded with a modified Renishaw InVia spectrometer using 785 nm excitation.^39^

Dispersion corrected periodic density functional theory (DFT-D) calculations were carried out with CASTEP (version 17.21).^40^ On-the-fly generated norm conserving pseudopotentials with a plane-wave cut-off of 870 eV were used with the PBE^41^ functional with the Tkatchenko–Scheffler (TS) dispersion correction scheme^42^ within the generalized gradient approximation (GGA). Brillouin zone sampling of electronic states was performed on a 10 × 8 × 9 Monkhorst–Pack grid (180 *k*-points). The starting structure was that determined here at 5 K. The equilibrium structure, an essential prerequisite for lattice dynamics calculations, was obtained by BFGS geometry optimization after which the residual forces were converged to |0.00097| eV Å^−1^. A second calculation that optimised both the lattice parameters and the geometry resulted in a 5.9% increase in the volume of the unit cell. Phonon frequencies were obtained by diagonalization of the dynamical matrix, computed using density-functional perturbation theory,^43^ to compute the dielectric response and the Born effective charges, and, from these, the mode oscillator strength tensor and infrared absorptivity were calculated. Raman intensities were calculated by a finite displacement method.^44^ In addition to the calculation of transition energies at zero wavevector, phonon dispersion was also calculated along high symmetry directions throughout the Brillouin zone. For this purpose, dynamical matrices were computed on a regular grid of wavevectors throughout the Brillouin zone, and Fourier interpolation was used to extend the computed grid to the desired fine set of points along the high-symmetry paths.^45^ The atomic displacements in each mode, that are part of the CASTEP output, enable visualization of the modes in Materials Studio^46^ to aid assignments and are also all that is required to generate the INS spectrum using the program ACLIMAX (version 6.0.0 LE).^47^ It is emphasised that, for the calculated spectra and dispersion curves shown, the transition energies have not been scaled.

## Conclusions

In this work we have shown that the structure of cryolite is the same at 5 K as previously determined at room temperature.^25–27^ We have used a combination of vibrational spectroscopies to observe all of the modes for the first time, including those that are infrared and Raman forbidden under octahedral symmetry.

This work also allows some assignments to be made for elpasolite^20^ and Cs_2_Na[AlF_6_].^21^ For the former, the Raman bands at 561, 330 and 138 cm^−1^ are *ν*_1_, *ν*_5_ and the librational mode, the infrared bands at 590, 401 and 238 cm^−1^ are *ν*_3_, *ν*_4_ and a Na^+^ translation. For Cs_2_Na[AlF_6_] the Raman bands at 520 and 364 cm^−1^ are *ν*_1_ and *ν*_2_, those at 316, 310 and 307 cm^−1^ are from *ν*_5_.

## Conflicts of interest

The authors declare no conflict of interest.

## Supplementary Material
